# Dietary Fat and Cancer—Which Is Good, Which Is Bad, and the Body of Evidence

**DOI:** 10.3390/ijms21114114

**Published:** 2020-06-09

**Authors:** Bianka Bojková, Pawel J. Winklewski, Magdalena Wszedybyl-Winklewska

**Affiliations:** 1 Department of Animal Physiology, Institute of Biology and Ecology, Faculty of Science, P.J. Šafárik University in Košice, 041 54 Košice, Slovakia; bianka.bojkova@upjs.sk; 2 Department of Human Physiology, Medical University of Gdansk, 80-210 Gdansk, Poland; magdalenawinklewska@gumed.edu.pl; 3 Department of Anatomy and Physiology, Pomeranian University of Slupsk, 76-200 Slupsk, Poland

**Keywords:** high-fat diet, cancer, inflammation, oxidative stress, saturated fatty acids, unsaturated fatty acids, trans fatty acids

## Abstract

A high-fat diet (HFD) induces changes in gut microbiota leading to activation of pro-inflammatory pathways, and obesity, as a consequence of overnutrition, exacerbates inflammation, a known risk factor not only for cancer. However, experimental data showed that the composition of dietary fat has a greater impact on the pathogenesis of cancer than the total fat content in isocaloric diets. Similarly, human studies did not prove that a decrease in total fat intake is an effective strategy to combat cancer. Saturated fat has long been considered as harmful, but the current consensus is that moderate intake of saturated fatty acids (SFAs), including palmitic acid (PA), does not pose a health risk within a balanced diet. In regard to monounsaturated fat, plant sources are recommended. The consumption of plant monounsaturated fatty acids (MUFAs), particularly from olive oil, has been associated with lower cancer risk. Similarly, the replacement of animal MUFAs with plant MUFAs decreased cancer mortality. The impact of polyunsaturated fatty acids (PUFAs) on cancer risk depends on the ratio between ω-6 and ω-3 PUFAs. In vivo data showed stimulatory effects of ω-6 PUFAs on tumour growth while ω-3 PUFAs were protective, but the results of human studies were not as promising as indicated in preclinical reports. As for trans FAs (TFAs), experimental data mostly showed opposite effects of industrially produced and natural TFAs, with the latter being protective against cancer progression, but human data are mixed, and no clear conclusion can be made. Further studies are warranted to establish the role of FAs in the control of cell growth in order to find an effective strategy for cancer prevention/treatment.

## 1. Introduction

Traditionally, a high-fat diet (HFD) has been regarded as detrimental for health, but in many cases, it is the obesity as a consequence of excess caloric intake that is in the background of various pathologies, including diabetes, cardiovascular diseases, and cancer [1]. Excess of nutrients alters gut microbiota, which leads to activation of pro-inflammatory pathways, an increase in intestinal permeability and systemic inflammation [2,3,4]. An increase in reactive oxygen (ROS) and nitrogen species (RNS), which come from aerobic metabolism, hypertrophied adipocytes and monocytes/macrophages, leads to an overload of cellular antioxidant capacity and induction of oxidative stress, which exacerbates inflammation [5,6,7]. When evaluating the effect of dietary fat, it is necessary to focus not only on major classes of fatty acids (FAs) (saturated vs. unsaturated FAs) but also on different members of these classes, as preclinical data show that they may differ in activity and effects [8,9]. However, the evaluation of isolated effects of individual FAs is not possible in human studies, and in addition, the FAs spectrum in fats and oils varies, which complicates the analysis of the link to cancer and other diseases. This review briefly summarises underlying mechanisms of the link between fat and cancer, the role of dietary FAs in the signalling pathways involved in the regulation of cell proliferation and associations between major classes of FAs and the risk of cancer in experimental and human reports.

### Source of Data

We searched for papers in the PubMed and Scopus databases, using search terms, including “high-fat diet”, “total fat”, “fatty acids”, “saturated”, “monounsaturated”, “polyunsaturated”, “trans-fatty acids”, “cancer”, “obesity”, “inflammation”, “microbiota” and “dysbiosis”. Relevant studies published almost exclusively in the English language were retrieved. References were selected on the basis of relevance, importance, and novelty. Papers published in the past ten years were preferentially treated.

## 2. The Connection Between Fat, Gut Microbiota, and Inflammatory Diseases

**Figure 1:**
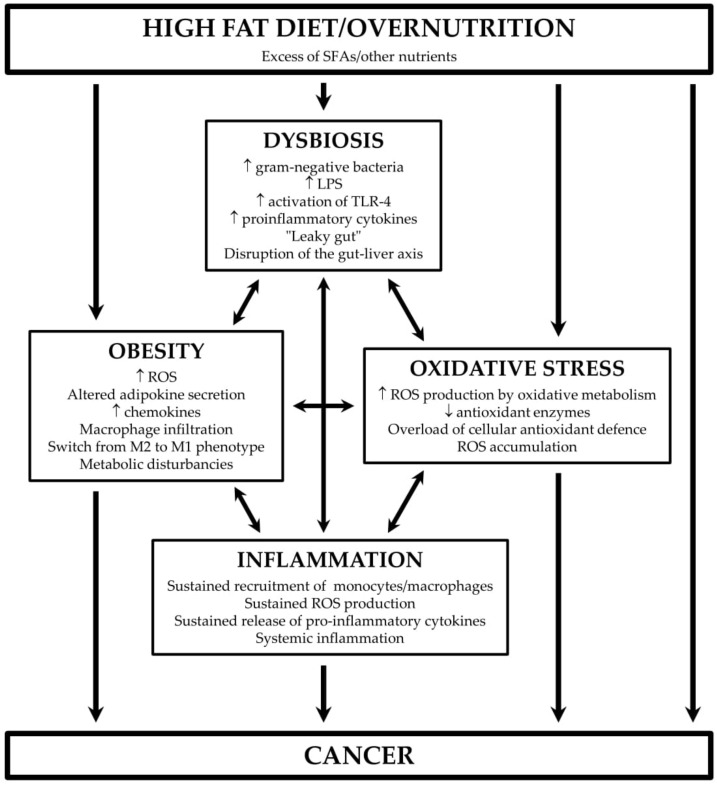
Interplay among disturbances induced by excess of nutrients. Abbreviations: LPS—lipopolysaccharides; ROS—reactive oxygen species; SFAs—saturated fatty acids; TLR-4—toll-like receptor 4.

Dietary lipids alter the microbiome, which plays a considerable role in the pathogenesis of many diseases, including cardiovascular disease, type-2 diabetes, and cancer [10,11]. Changes in the microbial community may be either beneficial or harmful to the host, depending on the lipid type. While ω-3 polyunsaturated fatty acids (PUFAs) seem to exert beneficial effects, saturated FAs (SFAs) were proved to promote dysbiosis. In mice fed with an HFD rich in saturated fat (lard) in comparison with those fed with an HFD containing ω-3 PUFAs (fish oil), the phylogenetic diversity and the abundance of beneficial intestinal bacteria was lower [12]. Similar results were reported in other murine studies [13,14]. HFD supplemented with palm oil, a source of SFAs, shifted the intestinal microbiota population to one similar to that seen in an obese phenotype, while an HFD supplemented with flaxseed/fish oil increased the intestinal levels of beneficial bifidobacteria [13]. A diet high in saturated but not in ω-6 PUFAs increased gut permeability and induced colonic inflammation and mesenteric fat inflammation in mice. On the other hand, the addition of ω-3 PUFAs to a diet rich in saturated fat showed a tendency to increase transepithelial resistance of the colon in the same study [14]. Changes of the gut microbiota induced by long-term administration of HFD to mice were associated with increased intestinal ROS production and oxidative stress [15], which play a significant role in cancer initiation and progression [16] (Figure 1).

Variations in gut microbiota also depend on host genetics, but according to an extensive study by Carmody et al. [17], diet plays the dominant role. Carmody and his co-workers evaluated the effect of an HFD and a high-sugar diet in five inbred mouse strains, four transgenic lines (mice deficient for genes relevant to host-microbial interactions) and in outbred strains and found that gut microbiota was reproducibly altered despite differences in host genotype. However, most changes to the gut microbiota were reversible, as revealed by repeated dietary shifts [17].

The adverse effect of a saturated fat-rich diet on gut microbiota and the overall host metabolic effect has been extensively studied, and the induction of chronic low-grade inflammation is presumed as the underlying mechanism (reviewed in [18,19,20]) (Figure 1). An HFD increases the abundance of Gram-negative bacteria [12,21,22], which contain lipopolysaccharides (LPS) on their outer membrane. The lipid A component (or endotoxin) of LPS binds to toll-like receptor 4 (TLR-4) [23], leading to the activation of nuclear factor kappa B (NF-κB) signalling and release of pro-inflammatory cytokines [24]. TLR-4 may also be stimulated directly by free FAs [25]. In addition, an HFD increases barrier-disrupting cytokines (tumour necrosis factor alpha [TNFα], interleukin [IL] 1B, IL6, and interferon γ) and decreases barrier-forming cytokines (IL10, IL17, and IL22) [26]. All this leads to an increase in gut permeability, which promotes the passage of LPS, free FAs, and pro-inflammatory cytokines into the circulation. As a result, systemic inflammation arises, which is a known risk factor for numerous diseases, including cardiovascular diseases, type-2 diabetes, and cancer [2,3,4] (Figure 1). Loss of microbial diversity, followed by increased endotoxin levels and increased intestinal permeability caused by gut inflammation, was also reported after high-glucose and high-fructose diets [27]. 

HFD promotes a decrease in Bacteroidetes and an increase in Firmicutes and Proteobacteria, the first two being the major bacterial phyla in the human intestine [4]. Increased consumption of saturated fat from meat and other animal foods also increases the intake of choline and L-carnitine, which are converted to trimethylamine (TMA) by intestinal bacteria. TMA-producing bacteria largely belong to the Firmicutes and Proteobacteria phyla, the enzymes required for this conversion are absent in Bacteroidetes. TMA is transported to the liver and metabolised into trimethylamine-N-oxide (TMAO), which has been linked to cancer via inflammation induction; other suggested mechanisms include oxidative stress, DNA damage, and disruption in protein folding. Plasma TMAO levels were positively correlated with the risks of various cancers (reviewed in [28]). Thus, the shift in Firmicutes to Bacteroidetes ratio in favour of Firmicutes, may result in increased TMAO production and contribute to cancer risk.

Not all LPS act strictly as immunostimulants. A human report showed that the total LPS derived from the gut microbiome of healthy adults inhibits TLR-4 signalling, and these immune silencing properties were attributed to the species of the order Bacteroidales [29]. Thus, the immunogenicity of gut microbial communities is determined mostly by the composition of the microbiota and subsequent LPS isoforms [30].

An increase in intestinal permeability and development of inflammation promotes obesity, which, in turn, contributes to an oxidative stress and inflammatory state, via increased ROS production in adipocytes [31] and “leaky gut” (Figure 1). A murine study showed that obesity increased the expression of cell death and cell survival/proliferation genes, which were associated with an increase in intestinal permeability, alterations in villi/crypt length, and decrease of tight junctions, and mucus synthesis. However, the authors acknowledged it was not clear whether obesity or gut dysbiosis contributed primarily to these changes [32]. In obese subjects, circulating levels of zonulin, a marker of intestinal permeability, were increased proportionally to daily energy intake [33]. It seems, though, that the type of body fat matters, as Gummesson et al. [34] reported that increased intestinal permeability in normal to overweight women was associated with visceral adiposity but not total body fat or subcutaneous fat [34].

Bacterial elements transported in the bloodstream activate TLRs and NF-κB signalling in the liver. In turn, both immune and hepatic non-immune cells, such as hepatic stellate cells and endothelial cells, release a set of pro-inflammatory cytokines, including TNFα, IL-6, and IL-1β [35,36]. As a consequence, sterile inflammation results in disruption of the lobular architecture and nodular reorganization, and subsequent fibrogenesis, the typical features of non-alcoholic and alcoholic liver disease [37,38]. Importantly, increased gut permeability and the consequent augmented presence of bacterial products in the bloodstream triggers a response in the liver, increasing the portal pressure, with the latter resulting in intestinal oedema, disruption of epithelial integrity, and more translocation from gut to the blood, creating a vicious circle [39]. The disruption of the gut–liver axis by dietary fat thus plays an important role in the development and progression of portal hypertension, chronic liver disease, and cirrhosis, with the latter actually being a pre-cancer state [40] (Figure 1).

Both animal and human reports focus almost exclusively on the bacterial component of the microbiome, but apart from the bacteria, the human microbiome also hosts viruses, fungi, and archaea that can all be altered in disease states [41]. In a murine study, an HFD significantly altered six fungal taxa abundances along with 16 bacterial taxa. These results suggest that the role of microbiome components may be interconnected [42], which should be considered in an evaluation of the impact of dietary fat on the pathogenesis of the human disease.

## 3. HFD, Oxidative Stress, and Inflammation

ROS (e.g., oxygen-free radicals, hydrogen peroxide and lipid peroxides) and RNS (e.g., nitric oxide, peroxynitrite), are generated as a by-product of aerobic metabolism and are necessary for many physiological processes, including cell differentiation, apoptosis, immunity and reproduction [43,44,45]. ROS/RNS may bind with membrane lipids, nucleic acids, proteins, enzymes, and other small molecules. ROS/RNS are highly reactive and can damage cell structures and alter their functions, but aerobic organisms possess both enzymatic and non-enzymatic antioxidant systems, which are usually effective in blocking their harmful effects. However, overproduction of ROS/RNS in pathological conditions may overwhelm these protective systems, which leads to the shift of the balance between oxidants and antioxidants in favour of oxidants, referred to as oxidative stress [46]. Oxidative stress may activate various transcription factors, including those that regulate the expression of genes involved in inflammatory pathways [16,47] (Figure 1).

Lipid accumulation in adipocytes induced by HFD leads to their hypertrophy and to changes in adipokine secretion. Hypertrophied adipocytes contribute to the production of ROS [48], which induces the release of pro-inflammatory cytokines, including the monocyte chemoattractant protein-1 (MCP-1) [31], and, vice versa, pro-inflammatory cytokines promote an increase in ROS generation by macrophages and monocytes [49]. It is presumed that the source of the ROS differs during the course of obesity—in the early stage, ROS are generated by nicotinamide adenine dinucleotide phosphate oxidase (NOX) in adipocytes, followed by NOX-generation in macrophages and transiting to mitochondrial production in late stages of obesity [50]. The accumulation of ROS is facilitated by decreased expression of antioxidant enzymes [31,51]. The expression of pro-inflammatory cytokines is higher in visceral fat in comparison with subcutaneous fat tissue and is further enhanced in obesity [52]. Enhanced chemokine secretion, adipocyte death, hypoxia, and increased FAs flux, driven by hypertrophy, lead to an initiation of macrophage infiltration [53]. Preferential macrophage infiltration into omental fat vs. subcutaneous fat was reported in lean subjects and was exaggerated in obesity [54]. Obesity also induces a phenotypic switch of macrophages from an M2 anti-inflammatory to an M1 pro-inflammatory state [55], a crucial role in the regulation of the M1 phenotype is attributed to ROS [56]. As a result of these events, a positive feedback-loop between inflammation and oxidative stress in obese adipose tissue is established [5,6,7] (Figure 1).

Obesity can affect other tissues via the excess of free FAs, pro-inflammatory factors, and altered adipokine production and may lead to the development of insulin resistance, dyslipidemia, non-alcoholic fatty liver disease, and other metabolic disturbances [57]. Chronic low-grade systemic inflammation associated with HFD and obesity is involved in the pathogenesis of many diseases, including type-2 diabetes, cardiovascular diseases, intestinal diseases, chronic kidney diseases, osteoporosis, central nervous system disorders, and cancer [2]. As visceral fat is more metabolically active than subcutaneous fat, an increase in visceral adiposity poses a higher health risk compared to excess subcutaneous fat. Apart from metabolic syndrome, excess visceral adiposity was also associated with an increased risk of breast- [58], oesophageal- [59], and colorectal cancer [60], and chronic inflammation and alterations in adipokine production present another risk factor for tumourigenesis [61]. Furthermore, the fat distribution pattern may have an impact on survival and therapeutic response in several cancer types [62,63,64,65,66].

An HFD and obesity are associated with the activation of microglia [67,68] and astrocytes [67,69,70], leading to an inflammatory state in the brain. In particular, oxidative stress and low-grade systemic inflammation evoked by an HFD augments levels of TNF-α, IL-1β, IL-6, and inducible nitric oxide synthase in the rostral ventral lateral medulla (RVLM; [71]). Neuroinflammation in the RVLM promotes sympathetic nervous system activation and efferent transmission [71,72,73]. In neoplasms, the sympathetic overstimulation potentially occurs in both tumour cells and various elements of their microenvironment, i.e., lymphoid and myeloid immune cells, epithelial cells, adipocytes, fibroblasts, vascular myocytes, pericytes, glial and neural cells, [74]. The sympathetic nervous system may influence tumour β-adrenergic signalling both via circulating norepinephrine/epinephrine and via local norepinephrine release from sympathetic nerve fibres. Sympathetic fibres surround or even enter the tumour parenchyma in association with blood vessels. Such local release may provide neoplasm cells with higher neurotransmitter concentrations than those achieved in the bloodstream [75]. Importantly, the significant overexpression of adrenergic receptors has been reported in the variety of neoplasms, particularly in lymphoid tissues, bone marrow, kidney, adrenal glands, liver, stomach, colon, brain, lung, breast, ovary, prostate, skin and vasculature [74,76]. As the neoplastic process, regardless of location, is marked by systemic inflammatory response [77], HFD, cancer, and sympathetic nervous system may effectively create another vicious circle in neoplasm development [72,73,76,78]. Complex interactions between neoplasm growth and sympathetic nervous system were reviewed elsewhere in detail [75,79,80].

## 4. Xenobiotics in the Obesity-Cancer Link

An association between obesity and exposure to environmental contaminants, which can disrupt the normal developmental and homeostatic control over adipogenesis and energy balance and are referred to as obesogens, was suggested in 2006 [81]. Most of them are endocrine disrupters, interfering with the normal function of the endocrine system, and displaying carcinogenic properties [82]. For example, acrylamide, present in a wide range of heated foodstuffs, particularly in carbohydrate-rich foods [83], was shown to upregulate adipogenesis in mice via the increased expression of CCAAT-enhancer-binding proteins, which are adipogenic transcription factors for adipocyte differentiation. Acrylamide also induced phosphorylation of mitogen-activated protein kinases (MAPKs) and adenosine monophosphate-activated protein kinase (AMPK)-acetyl-CoA carboxylase, expression of adipocyte fatty acid-binding protein (aP2), lipoprotein lipase, sterol regulatory element-binding protein (SREBP)-1c and fatty acid synthase [84]. In a human report, acrylamide haemoglobin biomarkers in blood were associated with abdominal obesity as well as overweight [85]. We refer readers to excellent reviews by Newbold [86] and Heindel and Blumberg [87] for further information.

## 5. Mediterranean Diet and Cancer Risk 

The Mediterranean diet, or rather the lifestyle, is considered a powerful method to combat cancer. The positive effects of the Mediterranean diet have been widely reported [88], but there is no precise definition regarding the quantity and quality of components. A general description includes a high intake of monounsaturated FAs (MUFAs), from extra virgin oil, vegetables, fruits, legumes, cereals, and nuts, cutting meat and dairy consumption, and limited intake of sweets [89]. A comprehensive meta-analysis of 83 studies (total 2,130,753 subjects) evaluated the association between the Mediterranean diet and cancer risk and mortality. The highest adherence score to a Mediterranean diet was inversely associated with a lower risk of cancer mortality (relative risk [RR] _cohort_: 0.86, 95% confidence interval [CI] 0.81–0.91), colorectal cancer (RR_observational_: 0.82, 95% CI 0.75–0.88), breast cancer (RR _randomised controlled tria [RCT]_: 0.43, 95% CI 0.21–0.88) (RR_observational_: 0.92, 95% CI 0.87–0.96), gastric cancer (RR_observational_: 0.72, 95% CI 0.60–0.86), liver cancer (RR_observational_: 0.58, 95% CI 0.46–0.73), head and neck cancer (RR_observational_: 0.49, 95% CI 0.37–0.66) and prostate cancer (RR_observational_: 0.96, 95% CI 0.92–1.00) [90]. The Mediterranean diet, however, did not alter the risk of cancer mortality and recurrence among cancer survivors [90]. According to pooled analysis, the protective effects may be attributable to a higher intake of fruits, vegetables, and whole grains. However, as authors acknowledged, this report has some limitations, including varying dietary patterns, mixed exposure to carcinogens, methodological flaws of some of the studies, and lopsided availability of studies by type of cancer [90]. The recent analysis of 13 prospective cohort studies found an inverse relationship between the Mediterranean diet and bladder cancer risk [91]. Cancer-preventive properties of the Mediterranean diet may be attributed to several components. Antioxidants neutralise ROS/RNS, leading to a decreased rate in DNA mutations and downregulation of phosphatidylinositol 3-kinase (PI3K), MAPKs, and NF-κB proliferation pathways. Flavonoids contribute to antiproliferative effects and also attenuate the carcinogenic potential of xenobiotics by inhibition of some cytochrome P450 enzymes involved in the activation of pro-carcinogens and by induction of phase II detoxification enzymes. MUFAs and PUFAs downregulate NF-κB via peroxisome proliferator-activated receptor (PPAR) and exert anti-inflammatory effects. Dietary fibre attenuates post-prandial ROS/RNS peak and decreases the glycaemic index of foods, leading to a lower release of insulin and insulin-related growth factors. In addition, metabolisation of fibre by intestinal microbiota produces short-chain FAs that downregulate pro-inflammatory pathways, e.g., via G-protein-coupled receptor (GPR) family receptors (reviewed in [92]). The benefits of red wine intake remain controversial, as ethanol is classified as a human carcinogen [93], but on the other hand, red wine is a source of polyphenols and other beneficial substances that may counteract the carcinogenic effects of ethanol. Further, well-designed studies need to elucidate the potential of the Mediterranean diet in cancer prevention.

## 6. Dietary Fat and Cancer Risk

One of the earliest reports on tumour-promoting effects of fat in experimental cancer comes from 1930. Watson and Mellanby reported a higher incidence of tar-induced skin tumours in mice when butter was added to the diet [94]. A multitude of ensuing in vivo experiments mostly carried out in rodents, led to a general conclusion that HFD is positively associated with cancer risk. However, further analysis revealed that tumour incidence (involving several tumour sites in mice) was positively associated with total caloric intake, regardless of the level of dietary fat [95]. In addition, in isocaloric diets, it is the fat type that matters in tumour promotion, and progression [96,97,98], as the impact of different FAs on signalling pathways involved in cell proliferation varies [99,100]. Furthermore, interpolating animal data is tricky, in part because of differences in metabolism, in part due to factors which, unlike in vivo studies, cannot be controlled in full in human studies (e.g., total caloric intake, macronutrient, and micronutrient composition, lifestyle factors, etc.), at least not for a long time.

According to human studies, obesity, which is induced by both fat and carbohydrate-rich diet in most cases, has a higher impact on cancer risk than dietary fat content. In 2012, excess body weight accounted for almost 4% of all cancers globally and for 7–8% in some high-income Western countries and in Middle Eastern and Northern African countries [101,102]. A positive association between body mass index and cancer has been shown [103], but it appears that central adiposity is a stronger predictor of all-cancer risk than body size [104]. No such association may be unambiguously attributed to the total dietary fat [105]. Obesity is associated with redox and hormonal imbalances that promote tumour progression [106,107]; therefore, it must be considered when evaluating the role of fat composition in relation to cancer. The impact of different fats/FAs on tumourigenesis in preclinical and human studies is discussed in the next sections. The results of relevant studies are summarised in Table 1; we focused on data from the last 10 years.

### 6.1. Total Fat

The International Agency for Research on Cancer (IARC) in its European Code Against Cancer recommends limiting foods high in fat, but this advice is related to calorie surplus leading to excess body fat [108], which is linked to increased cancer risk at nine sites: oesophagus, colorectum, gall bladder, pancreas, breast (postmenopausal), endometrium, ovary, kidney, and prostate (advanced stage). It is estimated that 4–38% of these cancers (depending on site and gender) can be attributed to overweight/obesity [109]. As previously mentioned, low-grade systemic inflammation caused by excess adiposity, especially visceral adiposity, may be the underlying cause.

Epidemiological data do not support the hypothesis that a mere decrease in total fat intake would be an effective way to prevent cancer [105,110,111,112,113] or decrease cancer-specific mortality [114]. On the other hand, it is possible that increased dietary fat content may alter visceral fat even if the energy intake is adequate. In animal reports, an HFD increased visceral adiposity in comparison with an isocaloric low-fat diet [115,116]. Unluckily, human reports are scarce and contradictory. Isocaloric substitution of 5% of total energy from carbohydrates with fat was positively associated with visceral fat (and also hepatic fat but not subcutaneous fat) [117]. In another study, fat content in isocaloric diets (very high-fat, low-carbohydrate: 73% of energy fat and 10% of energy carbohydrate vs. low-fat, high-carbohydrate: 30% of energy fat and 53% of energy carbohydrate) did not alter visceral adiposity, but this report included a smaller number of participants and a shorter intervention period [118]. Proper evaluation of the role of fat in isocaloric diets requires unified methodology, including the definition of the FAs spectrum.

The impact of dietary fat depends not only on quality and quantity but also on a number of other factors, including the host genetics and the gender [119]. General advice of nutritionists is to prefer plant products and cut down on animal fat intake from meat, particularly red meat, and dairy products due to the high content of saturated fat. However, animal products are also a source of essential nutrients, so excluding them completely may not always be the best choice. As mentioned above, it is the FAs spectrum that matters, as FAs differ in their biochemical properties and in their physiological and metabolic effects. Experimental data showed that some FAs might promote cancer independently of obesity, e.g., via the enhancement of progenitor cell stemness [120,121].

### 6.2. Saturated Fat

Promoting the effects of a diet high in saturated fats on tumourigenesis has been reported by many in vivo studies. In addition, it was also shown that this diet might compromise the inhibitory effect of anticancer treatment [122]. These effects are generally attributed to the major component of diets high in SFAs, palmitic acid (PA), which is discussed below. However, neither experimental nor human reports are unambiguous regarding the tumour-promoting properties of saturated fat per se. Saturated fat intake was associated with higher cancer mortality (highest vs. lowest quintile [Q5 vs. Q1]: HR: 1.26, 95% CI 1.20–1.32) in a prospective cohort study of 521,120 participants, with 16 years of follow-up [123]. High intake of saturated fat (but not total, monounsaturated or polyunsaturated fat intake) was associated with increased risk of breast cancer (Q5 vs. Q1: hazard ratio [HR]: 1.13, 95% CI 1.00–1.27) in a large European multicentre prospective study (519,978 participants) [103] and also in a recent French prospective study (Q5 vs. Q1: HR: 1.98, 95% CI 1.24–3.17); the latter also linked the saturated fat to increased overall cancer risk (Q5 vs. Q: HR: 1.44, 95% CI 1.10–1.87) [124]. Similarly, breast cancer survival was negatively affected by saturated fat in a meta-analysis of cohort studies (highest vs. lowest category of intake: HR: 1.51, 95% CI 1.09–2.09) [114]. SFAs intake was associated with an increased risk of prostate cancer, too (HR: 1.19, 95% CI 1.07–1.32) [125]. However, a meta-analysis of prospective cohort studies did not show an association between SFAs intake and colon cancer risk; the intake of MUFAs, PUFAs, or total fat did not have any impact either [112]. No associations were observed in the subgroup analyses of dietary SFAs, MUFAs, and PUFAs intake, and epithelial ovarian cancer risk [126]. Interestingly, several case-control studies reported statistically significant or borderline decreased risks of pancreatic cancer with a higher saturated fat intake (summarised in [127]).

Research data showed that factors other than saturated fat content must be considered. For example, the association between consumption of red and processed meat and the risk of colorectal cancer [128] may be explained by the formation of carcinogenic heterocyclic amines and polycyclic aromatic hydrocarbons during the cooking process. Another factor is the generation of lipid oxidation products and nitroso compounds catalysed by haem-iron during digestion. The risk is also modulated by the effect of food processing-borne xenobiotics on the gut microbiota [129,130,131]. According to IARC and World Cancer Research Fund/American Institute for Cancer Research, red meat consumption may increase the risk of lung, pancreatic, and prostate cancers [132]. The general recommendation for reducing colon cancer and other cancers risk via a healthy diet is to cut the intake of red and processed meat, refined grains, sweets, caloric drinks, juices, convenience food, and sauces and stick to Mediterranean patterns of diet, preferring consumption of whole fruits, vegetables, legumes, olive oil, nuts, and fish [133].

The association between saturated fat in dairy products and cancer risk is not clearly established, due to methodologic limitations of most studies [134]. A large U.S. population-based cohort study and meta-analysis found no link between total dairy consumption and risk of cancer or cancer mortality. However, a recent meta-analysis of observational studies found that the risk of ovarian cancer was increased in non-linear form for both saturated and monounsaturated fat from 25 g/day [135]. Fermented dairy product consumption was inversely related with total mortality (RR: 0.97, 95% CI: 0.96–0.99) but not cancer mortality [136]. A recent prospective study, however, found a positive association between total intake of dairy products (highest vs. lowest tertile [T3 vs. T1]: HR: 1.85, 95% CI 1.19–2.88) and intake of high-fat dairy products (HR: 1.81, 95% CI 1.19–2.76) and hepatocellular carcinoma risk. Yogurt consumption showed a non-significant inverse association with hepatocarcinoma risk (HR: 0.72, 95% CI 0.49–1.05) [137]. It is necessary to remark that different outcomes of studies may be attributed to not only differences in study design but also to the way animals are raised, as it has an impact on the levels of various PUFAs and inflammatory factors in food products [138].

#### 6.2.1. The Role of SFAs in Signalling Pathways Involved in Cancer

##### Palmitic Acid

Understanding the role of fat in carcinogenesis requires elucidation of the role of FAs in signalling pathways involved in cell proliferation. The research in this area is ongoing, and, among SFAs, PA has drawn great attention. Palm oil, which contains about 44% of PA [139], is, in general, the major source of PA in the human diet, but PA is also present in high quantities in other oils and fats, accounting for approximately 28% in butter, 27% in lard, 27% in beef tallow, 13% in corn oil and, 10% in olive oil [140]. The estimated average daily intake is 20–30 g corresponding to 8–10 energy % (according to the Third Italian National Food Consumption Survey [141]). PA is the most abundant SFA in the human body and can be provided in the diet or synthesised endogenously from other FAs, carbohydrates, and amino acids. PA accounts for 20–30% of total FAs in membrane phospholipids, adipose triacylglycerols and breast milk [142].

There is increasing evidence that PA acts as an intracellular signalling molecule and is involved in the pathogenesis of cancer and other diseases, including metabolic syndrome, cardiovascular and neurodegenerative diseases, and inflammation [143]. PA participates in post-translational modifications of proteins, a process called S-palmitoylation, when PA is linked to the proteins by a thioester bond, catalysed by 23Asp-His-His-Cys (DHHC)-family palmitoyl S-acyltransferases, while the removal of PA is catalysed by serine hydrolases, including acyl-protein thioesterases. Palmitoylation functions as a switch regulating protein’s function. Palmitoylation regulates the functions of many proteins involved in homeostasis, e.g., G-protein coupled receptor. The dysregulation of protein function by palmitoylation contributes to metabolic disorders, neuronal diseases, and also cancer [143,144,145]. Palmitoylation is essential for the function of both oncogenes (e.g., *HRAS*, *NRAS*, and epidermal growth factor receptor [*EGFR*]) and tumour suppressors (e.g., *SCRIB*, melanocortin 1 receptor) [145,146].

Preclinical data showed both stimulatory and inhibitory effects of PA on tumour growth. PA increased the proliferation of colorectal cancer cells in a β2-adrenergic receptor (AR)-dependent manner. The stimulatory effect of an HFD, which increases the levels of PA and stearic acid (SA), or PA-rich diet on the growth of HCT116 colorectal cancer cells was abolished in mice bearing β2-adrenergic receptor (AR)-knockout xenografts [8]. Ex vivo murine data showed an increase in murine *Lgr^5+^* intestinal stem-cells in a PPAR delta-dependent manner after PA treatment [121]. In PNT1A and PC3 prostate cancer cell lines, PA promoted cell migration via vimentin expression and increased the levels of activated extracellular signal-regulated kinase 1/2 (ERK1/2), leading to increased proliferation, despite activation of AMPK [147]. PA upregulated the biosynthesis of palmitoyl-CoA in PC-3 prostate cancer cells in vitro and in vivo. A diet high in PA enhanced the proliferation of prostate cancer xenografts in comparison with a diet high in unsaturated fat. PA increased the level of Src kinase and Src-mediated downstream signalling, including MAPK activation, and also enhanced Src-dependent mitochondrial β-oxidation [98]. PA increased the invasiveness of AsPC-1 pancreatic cancer cells via the TLR-4/ROS/NF-κB/matrix metalloproteinase-9 (MMP-9) signalling pathway [148] and promoted metastasis in several human oral carcinoma cell lines expressing high levels of cluster of differentiation 36 (CD36) [149]. The tumour promoting effect of PA via CD36 was reported in gastric cancer cells too, and PA induced metastasis by phosphorylation of protein kinase B (AKT), leading to activation of AKT/ glycogen synthase kinase-3 beta (GSK-3β)/β-catenin signalling pathway [150]. On the other hand, PA induced cell cycle delay and CCAAT-enhancer-binding protein homologous protein (CHOP) dependent apoptosis and was also involved in activation of the endoplasmic reticulum (ER) stress response network via X-box activating protein 1 (XBP1) and activating transcription factor 6 (ATF6) in HER2/neu-positive breast cancer cells [151]. PA also inhibited proliferation, impaired cell invasiveness, and suppressed hepatocarcinoma growth in vitro and in mouse xenograft models, inhibition of the mammalian target of rapamycin (mTOR) and signal transducer and activator of transcription 3 (STAT3) pathway, decreased cell membrane fluidity, and impaired glucose metabolism was demonstrated [152]. PA was also reported to stabilise oncogenic protein beta-catenin in prostate cancer cells [153].

Results of human studies are not consistent, showing a positive association between dietary PA and breast (Q5 vs. Q1 HR: 1.68, 95% CI 1.13–2.50) [154] and prostate cancer (Q5 vs. Q1 RR: 1.53, 95% CI 1.07–2.20) or no association for both breast and prostate cancer risk [155]. Positive associations were found between circulating levels of plasma phospholipids PA and risk of breast (Q5 vs. Q1 HR: 1.86, 95% CI 1.27–2.72) [155] and prostate cancer ([HRs (Q5–Q2 vs. Q1] were significantly elevated) [156]. Elevated risk of prostate cancer for men with higher plasma levels of PA was found in another study too (Q5 vs. Q1: 1.47, 95% CI 0.97–2.23) [157]. A meta-analysis of two European studies showed a positive association of SFAs intake and epithelial ovarian cancer risk (highest vs. lowest quartile: overall HR: 1.21, 95% CI 1.04–1.41) [158]. On the other hand, a negative association between PA intake and pancreatic cancer risk was reported by Nkondjock (OR: 0.73, 95% CI 0.56–0.96) [127] (Table 1).

It must be emphasised that under physiological conditions, the changes in PA intake do not significantly alter its tissue concentration, which is maintained by endogenous biosynthesis from acetyl-CoA catalysed by acetyl-CoA carboxylase and fatty acid synthase. The homeostatic balance of PA may be disrupted by positive energy balance, excessive intake of carbohydrates, and a sedentary lifestyle, leading to overaccumulation of PA in tissues. This results in dyslipidaemia, hyperglycaemia, increased ectopic fat accumulation and increased inflammatory tone, and imbalance of PA/PUFAs ratio in the diet may contribute to these pathologies and promote cancer growth [142]. The current understanding is that the consumption of palm oil within a balanced diet does not pose a health risk (regarding cancer or cardiovascular disease) if SFA intake is kept under 10% of the total energy [159,160].

##### Stearic Acid

The isolated effect of other SFAs in tumourigenesis has been less studied. SA is found in large quantities, especially in cocoa butter but also in beef tallow, butterfat, and lard [140]. SA inhibited experimental breast cancer both in vitro and in vivo. SA inhibited the cell cycle at the G1 and G2 phases, increased cell cycle inhibitor p21*^CIP1/WAF1^* and p27*^KIP1^* levels, and decreased cyclin-dependent kinase 2 (CDK2) phosphorylation in Hs578T human breast cancer cells. SA also inhibited Rho activation and expression. These results were confirmed in vivo too, and dietary stearate decreased Rho expression in rat mammary tumours induced by N-methyl-N-nitrosourea (NMU) [161].

SA reduced visceral adiposity in athymic nude mice by the promotion of apoptosis via increased caspase-3 activity, decreased cellular inhibitor of apoptosis protein-2, and increased *Bax* expression in preadipocytes, although it did not alter differentiation or the viability of mature adipocytes. On the contrary, oleic acid (OA) and linoleic acid (LA) showed no apoptotic effects [162].

Human results are contradictory. A cohort study found a positive association between SA intake and breast cancer risk (Q5 vs. Q1 HR: 1.65, 95% CI 1.12–2.43) [154]. On the contrary, SA intake was associated with a decreased risk for pancreatic cancer (OR: 0.70, 95% CI 0.51–0.94) [127] (Table 1).

Interestingly, recent data indicate that SA may be useful in cancer treatment in a form other than the dietary intervention. A sialic acid-SA conjugate nanocomplexes with encapsulated ibrutinib, which is an inhibitor of Bruton’s tyrosine kinase, effectively targeted tumour-associated macrophages both in vivo and in vitro, resulting in inhibition of tumourigenic cytokine release, reduction of angiogenesis and growth suppression of S180 murine sarcoma [163]. This and other papers [164,165,166] indicate that the use of SA and also other SFAs in lipid-based nanoparticles is a promising strategy for targeted cancer therapy.

##### Lauric Acid (LaA)

LaA, the dominant SFA in coconut oil, showed antiproliferative and proapoptotic effects in human SkBr3 breast and Ishikawa endometrial cancer cells by upregulation of p21*^Cip1/WAF1^* in a p53-independent manner [167]. LaA also induced apoptosis in human colon cancer cells HCT-15 [9] and Caco-2 [168]; EGFR downregulation seemed to be an underlying mechanism [9]. LaA reduced cell proliferation, mitochondrial volume, and lactate production and increased oxidative stress in CT26 mouse colon cancer cells, particularly in low-glucose conditions, which indicates that it may reprogram the energy metabolism of cancer cells during glucose starvation [169] (Table 1). LaA improved sensitivity to cetuximab in *KRAS/BRAF* mutated colorectal cancer cells by induction of miRNA-378 expression [170]. The oncostatic effect may be increased by encapsulation to protect LaA from possible degradation in the extracellular environment [171]. LaA may also be used in a coating of nanoparticles to improve intracellular retention and drug delivery [172,173,174].

##### Myristic Acid (MA)

The major dietary source of MA is coconut oil and butter [140]. Myristoylation, an attachment of a myristoyl group to proteins by N-myristoyltransferase, typically occurs cotranslationally but also as a post-translational modification of proteins. Myristoylation, similar to palmitoylation, plays a significant role in regulating cellular signalling pathways in several biological processes, including carcinogenesis and immune function [175]. Myristoylation of Src kinase-mediated Src-induced and HFD accelerated progression of PC-3 prostate cancer xenografts in mice [122].

A positive association with prostate cancer was reported for MA intake in a Japanese cohort study (highest vs. lowest quartile RR: 1.62, 95% CI 1.15–2.29) [176], but plasma levels had no impact on prostate cancer risk in another study [155] (Table 1). We found no other reports regarding MA in human cancers.

Similar to other SFAs, MA has potential in cancer nanotherapy. Modification of DA7R peptide with an MA enhanced blood-brain barrier traversing efficiency of doxorubicin-loaded MA-DA7R liposomes, leading to high internalisation in glioma, tumour neovascular and brain capillary endothelial cells. Improvement of the glioma microenvironment resulted in a prominent therapeutic outcome in mice [177].

##### The Impact of Other SFAs on Carcinogenesis

There is limited data on the effects of SFAs that are only minor components of common fats and oils. Capric, caprylic, and caproic acids, which are present in goat milk (the first two also in coconut oil), reduced the viability of human HCT-116 colorectal, A-431 skin and MDA-MB-231 breast cancer cells in vitro by down-regulating cell cycle regulatory genes and up-regulating genes involved in apoptosis [178] (Table 1). Butyric acid, which is found in bovine milk but is also produced by microbial fermentation of fibre in the colon, showed tumour suppressive effects on colon cancer cells via histone deacetylase inhibitor (HDACi) activity. However, butyrate may also act independently of histone deacetylase (HDAC) inhibition, through the GPR109A receptor involved in inhibition of pro-inflammatory NF-κB signalling pathways. In addition, butyrate administration or dietary supplementation with resistant starches and other fermentable fibres had anti-obesogenic effects in rodents (reviewed in [179]). These results confirm the long-known beneficial effects of fibre in not only cancer prevention.

### 6.3. Unsaturated Fat

#### 6.3.1. MUFAs

Among MUFAs, OA is the most abundant representative in the human diet, accounting for more than 20% of all FAs in most of the common fats and oils, with the highest content in olive oil (approximately 78%) [140]. Preclinical data on OA effects on tumour promotion and progression are not consistent. OA enhanced the proliferation of breast carcinoma MCF-7 cells [180]. On the other hand, the treatment of human breast cancer cell lines BT-474 and SK-Br3 with OA suppressed *HER-2/neu* expression [181]. In another study, OA suppressed cell growth and survival in both MCF-7 and low metastatic gastric carcinoma cells SGC 7901, but this effect was restrained by pharmacological activation of AMPK, which rescued cell viability by increased beta-oxidation of FAs resulting in maintenance of ATP levels. In high-metastatic lines, HGC-27 and MDA-MB-231 treated with OA, AMPK was upregulated, which promoted cancer growth and migration [182]. The addition of OA nullified the inhibitory effects produced on MCF-7 and MDA-MB-231 cell migration by stearoyl-CoA desaturase-1 (SCD-1) depletion (pharmacological or siRNA-based) [183]. OA induced migration via free fatty acid receptors 1 and 4, promoted invasion through the PI3K/Akt pathway and increased NF-κB -DNA binding activity in MCF-7 and MDA-MB-231 cells [184]. The OA treatment enhanced the invasiveness of gastric cancer cell lines MKN-45 and AGS via activation of the PI3K-Akt signalling pathway [185]. OA promoted the growth of colon cancer cell line Caco-2 [186] and increased the invasiveness of 786-O renal cancer cells via an integrin-linked kinase pathway [187]. OA induced Src kinase and downstream ERK1/2 pathway activation in a CD36-dependent manner in He-La cells. A high olive oil diet aggravated growth and metastasising of He-La xenografts in mice; tumour progression correlated with CD36 expression [188]. On the other hand, OA induced apoptosis and autophagy in CAL27 and UM1 tongue squamous cell carcinomas by blocking the Akt/mTOR pathway [189].

Altogether, most of the experimental data show the growth-stimulatory effects of OA on cancer cells. However, olive oil contains a number of bioactive substances, including polyphenols and triterpenes, with anti-tumoural, anti-inflammatory, and antioxidant properties [190]. Cultivation of colon cancer cells with hydroxytyrosol, oleuropein, pinoresinol, squalene and maslinic acid (0.1–10 μM) reverted DNA synthesis, and Caco-2 cell growth induced by OA in a previously mentioned study [186]. Thus, the impact of OA on cell proliferation may be counteracted by these minor components, and the results of human studies support this hypothesis.

A meta-analysis of case-control studies showed that olive oil consumption was associated with lower odds of having any type of cancer (highest vs. lowest intake category; log odds ratio [OR] = −0.41, 95% CI −0.53 to −0.29). Moreover, olive oil consumption was associated with lower odds of developing breast cancer (logOR = −0,45, 95% CI −0.78 to −0.12) and cancer of the digestive system (logOR = −0,36, 95% CI −0.50 to −0.21). As the authors acknowledged, it is not clear whether the beneficial effects of olive oil may be attributed to MUFAs content or to antioxidant content [191]. MUFAs intake was also inversely associated with a decreased risk of digestive cancers in another study (Q5 vs. Q1 HR: 0.41, 95% CI 0.18–0.95) [124]. A meta-analysis of observational studies found an inverse association between MUFAs consumption and the risk of basal cell carcinoma (RR: 0.90, 95% CI 0.85–0.96) [113]. However, an increased risk of prostate cancer with increasing intake of MUFAs was reported (HR per Q: 1.14, 95% CI 1.03–1.27) [125].

Isocaloric replacement of MUFAs from animal sources with MUFAs from plant sources decreased cancer mortality in two prospective cohort studies (HR: 0.73, 95% CI 0.65–0.82) [192]. In another prospective cohort study with 16 years of follow-up (521,120 individuals), isocaloric replacement of 5% of the energy from SFA with plant MUFAs was associated with an 11% decrease in cancer mortality (HR: 0.89, 95% CI 0.83–0.95) [123] (Table 1). These results point to the beneficial effects of MUFAs from plant sources. The effect of individual sources of plant MUFAs was not analysed in these studies, but nevertheless, according to a consensus report of the 3^rd^ International Conference on Virgin Olive Oil and Health held in Spain in 2018, substantial evidence supports the widespread opinion that extra virgin olive oil should be the fat of choice when it comes to human health [193].

Nuts as another source of MUFAs may decrease cancer risk. Many nuts contain mostly MUFAs (mainly OA), and Brazil nuts have similar proportions of MUFAs and PUFAs, whereas walnut contains mainly PUFAs, both LA and alpha-linolenic acid (ALA). Moreover, the beneficial effects might be attributed to antioxidants and other phytochemicals and the fibre [194]. An inverse association between nut consumption and cancer was found for colorectal cancer for the ≥3 servings per week group vs. none (for women: adjusted ORs: 0.30, 95% CI 0.15–0.60; for men: adjusted ORs: 0.28, 95% CI 0.17–0.47) [195]. Decreased rates associated with nut consumption, even if not significant, were reported in relation to pancreatic cancer (highest intake vs. non-consumers: HR: 0.89, 95% CI 0.72–1.10) in the European Prospective Investigation into Cancer and Nutrition (EPIC) study with 476,160 participants and mean follow-up of 14 years [196]. These findings encourage the preference for plant sources of fat.

#### 6.3.2. PUFAs

There are two main groups of biologically significant PUFAs: omega-6 PUFAs (ω-6 PUFAs) and ω-3 PUFAs, classified according to the location of the first unsaturated bond. LA, a ω-6 PUFA, is the dominant PUFA in dietary fats and oils, except for flaxseed oil. The most common ω-3 PUFA is ALA, which can be found in the highest quantities in flaxseed oil (approximately 55% of the total FAs content) [197]; other dietary sources of PUFAs include soybean oil, canola oil and fish [140]. Both LA and ALA are essential FAs for humans, and they give rise to arachidonic acid (AA, ω−6), eicosapentaenoic acid (EPA, ω−3) ], and docosahexaenoic acid (DHA, ω−3), which play key roles in regulating body homeostasis [198]. In humans, DHA and EPA are predominantly acquired in the diet (mainly from fish oil) because the efficacy of transforming ALA to longer ω-3 PUFAs is low and personally variable [199].

In general, AA is a precursor to pro-inflammatory eicosanoids, whereas EPA and DHA are precursors to anti-inflammatory eicosanoids [200], but the interaction of ω-3 and ω-6 FAs and their lipid mediators in the context of inflammation is complex and yet not fully understood [201]. Still, the maintaining of a low ω-6/ω-3 ratio has been considered crucial for reducing inflammation [202], which is a known risk factor for the number of chronic diseases. An ω-6 to ω-3 ratio of 4:1 is recommended, but in typical Western diets, the ratio is approximately 15:1 [203]. Differences in the dietary ω-6/ω-3 ratio may also be the reason for the increase in cancer rates, including breast and prostate cancer in Asian immigrants to the United States [204,205,206];.

##### Ω-6 PUFAs

In vitro reports brought mixed results regarding the effects of ω-6 PUFAs on cancer cell growth. AA inhibited the growth of T98G human glioblastoma cells [180]. AA and LA reduced cell proliferation and viability of PC-3 and C4-2 prostatic cancer cells [207] and also, in another study, in PC-3 and RWPE-1 prostate epithelial cells [208], but in this other study, the effect of LA was inhibitory only at high concentrations; lower concentrations stimulated cell growth [208]. Similarly, high concentrations of LA inhibited the growth of RKO and LOVO colon cancer cell lines, but the effect of low concentrations was stimulatory; the authors attributed cytotoxic effects of LA to enhanced ROS generation and decreased cellular antioxidant capacity [209]. The inhibition of cell proliferation and viability after exposure to LA in a concentration-dependent manner in SW480 and SW620 colon cancer cells was reported in another study too [210]. Inhibitory effects of LA on tumour growth were also reported in AGS human gastric epithelial adenocarcinoma cells; LA downregulated prostaglandin E2 synthesis, and telomerase activity [211]. On the contrary, LA promoted migration and invasion of MDA-MB-231 breast cancer cells; the reported mechanisms involved upregulation of phospholipase D [212], fascin [213], and activation of PI3K/Akt pathway [214]. LA promoted an epithelial-mesenchymal transition (EMT)-like process in MCF10A human mammary epithelial cells via downregulation of E-cadherin and upregulation of *Snail1*, *Snail2*, *Twist1*, *Twist2*, and *Sip1*, activation of focal adhesion kinase (FAK) and NF-κB and activation of MMP-2 and -9 secretions [215].

In vivo data point to stimulatory effects of ω-6 PUFAs on tumour growth. A high dietary ω-6/ω-3 ratio (15:1) increased tumour burden of oral carcinoma induced by 9,10-dimethyl-1,2-benz[a]-anthracene (DMBA) and betel quid extract in hamsters, with increased expressions of NF-κB, proliferating cell nuclear antigen (PCNA) and cyclin D1 in a buccal pouch in comparison with a normal (6:1) and low (2:1) ω-6/ω-3 ratio [216]. A high ω-6 fat diet induced earlier onset of pancreatic neoplasia in KRAS transgenic mice [217]. Dietary LA stimulated invasion and metastasis of OCUM-2MD3 human gastric cell carcinoma in mice through COX-catalysed metabolism and activation of ERK [218] and promoted angiogenesis in the same line by suppression of angiostatin through plasminogen activator inhibitor 1 [219]. Feeding mice with an ω-6 rich diet (44% energy from safflower oil containing 76% LA) led to increased *Cox-2* expression, epigenetic activation of *Ptsg-2* coupled with silencing of tumour suppressor *Apc* and accumulation of C-JUN and *Ccnd1*, thus contributing to colonic inflammation and cancer [220].

Ω-6 PUFAs may also modulate carcinogenesis via alteration of biotransformation. In DMBA-model of breast cancer, a diet enriched with ω-6 PUFAs (corn oil) upregulated Phase I enzymes prior to DMBA administration and raised the activity of CYP1s after induction while reducing the activity of Phase II enzymes, mainly quinone oxidoreductase 1, resulting in the higher formation of DMBA-DNA adducts in the mammary gland [221].

Human reports on the association between dietary PUFAs and cancer risk mostly focus on ω-3 PUFAs or the impact of ω-6/ω-3 ratio. A meta-analysis of prospective cohort studies from 2016 did not find an association between ω-6 PUFAs intake and breast cancer risk [110]; however, a recent analysis indicated that higher dietary intake ratio of ω-3/ω-6 is associated with a lower risk of breast cancer in Asian countries rather than in Western countries [222]. Isocaloric replacement of 2% of the energy from SFAs with LA was associated with an 8% decrease in cancer mortality (HR: 0.95, 95% CI 0.90–0.93) [123] (Table 1).

##### Ω-3 PUFAs

Positive effects of ω-3 PUFAs have been confirmed in numerous cancer cell lines. Physiological concentrations of ALA alone or combined with EPA and DHA reduced viability and expression of microRNA-21 in the MCF-7 mammary cancer cell line [223]. Most reports, however, focused on DHA because of its unique effect of altering membrane composition; DHA is considered as the major ω-3 PUFA involved in anticancer activity [224]. DHA treatment was associated with activation of caspase 1 and gasdermin D, increased IL-1β, and high mobility group box 1 protein (HMGB1) translocation towards the cytoplasm, as well as an increase in pore formation in MDA-MB-231 cells, altogether suggesting induction of pyroptosis [225]. Inhibition of proliferation by DHA in MCF-7 cells via pAKT signalling was found in another study [226]. DHA decreased the viability of HT-29 and CaCo-2 colorectal cancer cells and enhanced the effect of irradiation; the underlying mechanism involved the WNT/beta-catenin pathway [227]. Ω-3 PUFAs, particularly DHA, also modulated angiogenesis via miR-126 methylation and VEGF expression in HCT-116 and Caco-2 cells [228]. The oncostatic effects involved alterations of xenobiotic metabolism, downregulation and inhibition of CYP1 enzymes, resulting in reduced genotoxicity of benzo[a]pyrene in HT-29 and HCT-116 cells after exposure to EPA and DHA was reported [229]. Other mechanisms of colon carcinogenesis modulation by ω-3 PUFAs included the alteration of M2 macrophage polarisation during the inflammatory response [230]. DHA was also reported to enhance the oxaliplatin-induced decrease in cell viability and an increase in autophagy via ER stress/Sesn2 pathway in colorectal cancer cell lines both in vitro and in vivo [231]. DHA induced apoptosis of PANC-1 pancreatic cancer cells by suppressing the STAT3/ NF-κB -cyclin D1/survivin axis [232]. DHA inhibited proliferation and progression of A549 non-small cell lung cancer cells through ROS-mediated inactivation of the PI3K/Akt pathway [233], and also through the miR-138-5p/FOXC1 pathway in A549 and H1299 human lung cancer cell lines and LLC murine lung cancer cells [234]. Both EPA and DHA inhibited pancreatic cancer cell (SW1990 and PANC-1) growth in vitro and in vivo through downregulation of Wnt/beta-catenin signalling [235]. DHA reduced proliferation of the MHCC97L human metastatic hepatocarcinoma line through the inhibition of cyclin A/CDK2 [236]. EPA induced SKOV-3 ovarian cancer cells apoptosis via ERK1/2-mTOR- NF-κB pathways [237]. DHA and ALA decreased viability of ovarian cancer cells (SKOV3, A2780, HO8910), but only DHA also inhibited invasion and metastasis, via multiple molecular pathways [238]. Both DHA and EPA triggered G0/G1 arrest and induced apoptosis in LA-N-1 neuroblastoma cells [239]. DHA induced cell death via apoptosis and autophagy in several glioblastoma cell lines, both in vitro and in vivo [240].

Ω-3 PUFAs also showed anticancer effects in haematological malignancies. Both DHA and EPA induced a dose-dependent decrease in cell viability in five acute myeloid leukaemia cell lines; cell death was associated with the mitochondrial glycolytic switch and nuclear factor erythroid 2-related factor 2 (Nrf2) pathway activation [241]. DHA showed pro-apoptotic activity in Molt-4 acute lymphoblastic leukaemia cells, which was associated with p53 accumulation, survivin downregulation, and caspase-3 activation [242].

Dietary ω-3 PUFAs were effective in vivo too. Ω-3 enriched diet decreased proliferation and angiogenesis and increased apoptosis and tumour infiltration by immune cells in mice carrying 4T1 mammary tumour implants [243]. Increased dietary levels of ALA inhibited LM3 mammary tumour growth and metastasis in mice; tumours showed increased apoptosis and higher T-lymphocyte infiltration, with decreased expression of the oestrogen receptor α, while showing an opposite effect on the oestrogen receptor β [244]. Dietary ω-3 content may even decrease mammary cancer risk in offspring. Female offspring of mice fed with diet enriched with flaxseed oil or fish oil showed delayed puberty, and their mammary glands contained less terminal end buds, which are targets for malignant transformation. The incidence of DMBA-induced mammary tumours was lower in this offspring, and tumour cells showed reduced proliferation via inhibition of NF-κB and Jak-STAT pathways and increased apoptosis [245]. Ω-3 inhibited chemically-induced colorectal cancer via the prevention of the decrease of genomic DNA methylation in rats [246]. Fish-oil ω-3 PUFAs suppressed colorectal carcinoma growth in Apc^Min/+^ Mice, which correlated with CB1 receptor upregulation. CB1 receptor induction was associated with a concurrent inactivation of the Wnt/β-catenin pathway [247]. Ω-3 PUFAs enriched diet suppressed the growth of MC38 colorectal carcinoma in mice, and treatment of tumours with epoxydocosapentaenoic acids, metabolites of ω-3 PUFAs, reduced expressions of protooncogens *C-myc*, *Axin2* and *C-jun* in tumour tissues [248]. Ω-3 enriched diet with fish oil prevented pancreatic carcinoma in KRAS mice via AKT pathway inhibition [249]. A diet high in ALA from flaxseed oil inhibited t prostate cancer growth in *Pten*-knockout mice [250]. Dietary ω-3 PUFAs inhibited endometrial cancer xenografts growth in mice; the involved mechanism included the suppression of mTORC1/2 signalling [251,252].

In vitro and animal reports also showed that the incorporation of DHA in cell membranes improves drug uptake, thus the enhancing anticancer activity of chemotherapeutics [224]. Ω-3 PUFAs containing nanoparticles that are currently developed and tested showed multiple benefits for the prevention and cure of cancer, e.g., protection from degradation, increased bioavailability and delivery to target tissues, and thus, enhanced bioactivity [253].

The outcomes of human studies are not unambiguously positive. A possible reason may be that the anticancer effects of ω-3 PUFAs are dose-dependent; preclinical studies often use high concentrations, and the consumption of ω-3 PUFAs in most countries is too low for a positive outcome. In the case of breast cancer, the decreased risk was mostly found when the highest ω-3 PUFA consumption group was compared to the lowest ω-3 group or the highest ω-6 group, as the effect was counteracted by ω-6 intake [254]. A recent meta-analysis, however, found an inverse relation between fish ω-3 PUFAs consumption and breast cancer risk in Asian patients (OR: 0.80, 95% CI 0.73–0.87) [255]. Data from EPIC cohort study (521,324 participants, median follow-up 14.9 years) revealed an inverse association between long-chained ω-3 PUFAs and colorectal cancer risk (Q5 vs. Q1 HR: 0.86, 95% CI 0.78–0.95) [256]. Long-chain ω-3 PUFAs intake was associated with reduced endometrial cancer risk only in women with normal body mass index (observational study, 87,360 participants; HR: 0.59; 95% CI 0.40–0.82) [257] (Table 1). Consumption of ω-3 PUFAs does not seem to alter prostate cancer risk [258]. Nevertheless, ω-3 PUFA supplements are safe and were shown to improve the clinical outcome and prognosis of cancer patients, so they are potential candidates for multi-targeted cancer therapy [259] or, at least, for adjuvant therapy to ameliorate side effects of chemotherapeutics [260,261].

##### Trans Fatty Acids (TFAs)

TFAs are MUFAs or PUFAs with one or more double bonds in trans configuration. Naturally, TFAs are produced by bacterial metabolism of PUFAs in the rumen and are present in all fats from ruminants. However, industrially produced TFAs (iTFAs) are usually the major source of TFAs in the human diet; these are made by partial hydrogenation of vegetable or fish oils, and are used in a variety of food products. The most common representatives of ruminant TFAs (rTFAs) are conjugated linoleic acid (CLA), an isomer of LA, and vaccenic acid (trans-11 18:1, VA), which is metabolised to cis-9, trans-11 CLA in humans; the other rTFA is palmitoleic acid (t16:1n-7). Cis-9, trans-11-CLA (c9,t11-CLA) is the principal dietary form of CLA, but lower levels of the other isomers (t10, c12-CLA; t9, t11-CLA; and t10, t12-CLA) are also present in CLA food sources. Among iTFAs, elaidic acid (EA), the trans form of OA, is the dominant representative [262,263,264], but EA was found in small quantities in ruminant fat, too [265]. The concentration of rTFAs in ruminant fat is up to 6%, whereas the content of iTFAs in partially hydrogenated fat may be as high as 60% [263]. According to the World Health Organization recommendation, the total TFAs intake should not exceed 1% of the total energy intake [266]. A higher intake of TFAs is a known cardiovascular risk factor and may also be related to cancer risk too, but these effects are attributed to iTFAs [267]. Some rTFAs are beneficial, particularly CLA, which showed anticancer properties and also positive effects on obesity and atherosclerosis, both in preclinical and clinical studies [268]. The reported increased risk of some human cancers associated with rTFAs may be linked to high saturated fat content [269] (Table 1).

#### 6.3.3. iTFAs

Preclinical studies mostly showed stimulatory effects of the main iTFA, EA, on malignant transformation. EA enhanced growth and metastasis of CT26 and HT29 cells both in vitro and in vivo and also induced expressions of stemness factors CD133 and Oct14 [270] but did not stimulate DNA synthesis and growth of Caco-2 cells in another study [186]. Increased expression of stem cell markers, nucleostemin, and CD133, and the attenuation of anticancer effects of 5-fluorouracil after exposure to EA were observed in CT26 murine colorectal cells, the CMT93 murine rectal carcinoma cell line, and the LL2 murine lung cancer cell line too [271]. Oral administration of EA increased the metastasis of CT26 cells by upregulating stemness markers nucleostemin and CD133 [272]. Dietary EA increased DNA synthesis in Ehrlich tumour-bearing CBA mice and decreased their survival rate [273]. On the other hand, EA inhibited SH-SY5Y neuroblastoma cell growth and induced apoptosis by enhancing oxidative stress and activating the ER stress/ unfolded protein response (UPR) signalling pathway and the GRP78/ATF4/CHOP pathway [274] (Table 1). We found no reports on iTFAs effects on carcinogenesis in vivo.

#### 6.3.4. rTFAs

VA inhibited the proliferation of MCF-7 and SW480 cells, but the effect was dose-dependent and likely mediated by VA desaturation to c9,t11-CLA via delta9-desaturase [275]; growth inhibition was not reported in MCF-10A mammary cancer cells [276]. VA also suppressed the proliferation and induced the apoptosis of 5-8F and CNE-2 human nasopharyngeal carcinoma cells through a mitochondria-mediated apoptosis pathway [277].

The effects of CLA in tumourigenesis have been investigated by numerous studies, but not all have specified the type of isomer they used. It was reported that CLA isomers differ in their metabolic effects [262], and preclinical data show they may also exert different effects on cancer cell growth. CLA inhibited the growth of MCF-7 and MDA-MB-231 cells via oestrogen receptor α and PI3K/Akt pathway [278] and potentiated oncostatic activity of docetaxel in both lines [279]. C-9,t-11 CLA and t-10,c-12 CLA inhibited SCD activity in MCF-7 and MDA-MB-231 cells [280]. The growth inhibitory effect of three CLA isomers (c9,t11-CLA, t9,t11-CLA, and t10,c12-CLA) was investigated in MCF-7 breast cancer cells; among them, t9,t11-CLA was the most efficient isomer by decreasing MCF-7 proliferation, inhibiting migration, and inducing apoptosis [281]. In HCT-116 and HT-29 human colorectal carcinoma cells, t10,c12-CLA repressed cell proliferation and induced apoptosis, whereas c9,t11-CLA showed no effect on cell proliferation and apoptosis [282]. On the contrary, c9,t11-CLA, but not t10,c12-CLA inhibited cell migration and MMP-9 activity in SW480 colon cancer cells. Both isomers, though, suppressed metastasis of CT-29 xenografts in mice [283]. T10,c12 CLA suppressed proliferation and migration of SKOV-3 and A2780 ovarian cancer cells by inducing ER stress, autophagy, and the modulation of Src, while t9,c11 CLA did not attenuate the proliferation [284]. T-10,c-12 CLA inhibited the G1-S progression via p21 upregulation in DU145 human prostate carcinoma cells [285]. C9, t11- CLA induced apoptosis in RL 95-2 endometrial cancer cells independently of *Akt* [286].

CLA showed oncostatic effects in many animal reports, particularly in mammary cancer (reviewed in [287,288]; a c9,t11 isomer was mostly studied, and a dietary level of 1% CLA seemed to be the optimal dose for cancer inhibition in animal studies. However, the effect in gastrointestinal cancers was not consistent with the possible impact of the type of used isomer, and the conclusions regarding the impact on prostate cancer growth were contradictory (reviewed in [289,290]). A diet enriched with CLA administered to female rats corresponded to a lower susceptibility to DMBA-induced mammary tumours in their female offspring [291]. CLA also improved oncostatic efficacy of chemotherapeutics. A CLA-gemcitabine conjugate showed enhanced anti-tumour activity against MCF-7 cells and in mice carrying MCF-7 xenografts in comparison with unmodified gemcitabine [292]. B16-F10 melanoma growth was inhibited both in vitro and in vivo after treatment with iRGD-modified liposomes containing CLA and paclitaxel [293].

TFAs may alter carcinogenesis via inflammatory pathways too, but the reported data are controversial. TFAs, particularly VA and palmitoleic acid, inhibited the expression of inflammatory genes induced by TNF-α in human umbilical vein endothelial (HUVAC) cells and HepG2 hepatocarcinoma cells, independently of PPAR gamma activation. Interestingly, EA also decreased inflammatory gene expression in HUVEC but not in HepG2 cells in the same study [294]. On the contrary, promotion of pro-inflammatory signalling by trans isomers of EA, linoelaidic acid, and VA but not their corresponding cis-isomers was reported in vitro [295], and dietary EA promoted inflammation and oxidative stress in a mouse model of hyperlipidaemia [296].

Human reports brought mixed results. A recent meta-analysis did not find a link between CLA intake or total TFAs intake and risk of breast cancer [297]. A positive association between trans-fat intake and colon cancer was reported (highest quartiles of energy-adjusted TFAs consumption: RR: 1.45, 95% CI: 1.04–2.03, [298]; highest vs. lowest quartile OR: 1.37, 95% CI 1.10–1.71) [299]. However, the use of medication must be considered. High intake of TFAs slightly increased colon cancer in older subjects (≥ 67 years of age; OR: 1.4, 95% CI 0.9–2.1 for men; OR: 1.6, 95% CI 1.0–2.4 for women), but the concomitant use of non-steroidal anti-inflammatory drugs and hormonal replacement therapy decreased the risk [300]. A positive association between total TFA intake and prostate cancer was reported in a US cohort study (HR per Q: 1.21, 95% CI 1.08–1.35) [125], but a Norwegian cohort study found a negative association between vegetable TFA intake and pancreatic cancer in men (highest vs. lowest intake HR: 0.52, 95% CI 0.31–0.87) and non-Hodgkin lymphoma in both genders (HR: 0.70, 95% CI 0.50–0.98), and an inverse trend was observed for cancer of the central nervous system in women, too (HR: 0.58, 95% CI 0.32–1.04) [269]. Intake of fish TFAs was associated with a decreased risk of prostate cancer (HR: 0.82, 95% CI 0.69–0.96) and lung cancer in women (HR: 0.55, 95% CI 0.40–0.77); an inverse trend was reported for bladder cancer (HR: 0.76, 95% CI 0.56–1.02). On the other hand, fish TFAs increased the risk of rectal cancer (HR: 1.43, 95% CI 1.09–1.88) and multiple myeloma (HR: 2.02, 95% CI 1.24–3.28), and a positive trend was observed for stomach cancer, too (HR: 1.34, 95% CI 0.97–1.85). Ruminant TFAs were associated with a decreased risk of multiple myeloma (HR: 0.45, 95% CI 0.24–0.84) and malignant melanoma in women (HR: 0.57, 95% CI 0.32–1.02) but increased risk of non-Hodgkin lymphoma (HR: 1.47, 95% CI 1.06–2.04), non-melanoma skin cancer (HR: 1.54, 95% CI 1.02–2.33), cancer of mouth/pharynx (HR: 1.59, 95% CI 1.08–2.35) and post-menopausal breast cancer (HR: 1.17, 95% CI 0.91–1.49). As the authors concluded, increased cancer rates linked with rTFAs were possibly attributed to saturated fat intake [269]. A meta-analysis of prospective cohort studies (approximately 900,000 participants in total) found a significant association between TFA intake and the risk of ovarian cancer (overall RR: 1.25, 95% CI 1.08–1.44) [301]. Studies evaluating the serum levels of TFAs found a positive association between iTFAs and oestrogen-receptor negative breast cancer (T3 vs. T1 OR: 2.01, 95% CI 1.03–3.90) [302] and pancreatic cancer risk among men (OR T3 vs. T1: 3.00, 95% CI 1.13–7.99) [303] in the European Prospective Investigation into Cancer and Nutrition cohort (Table 1). These results indicate the different impacts of TFAs from different sources on cancer risk, but further research is warranted to elucidate the role of TFAs in human cancers.

**Table 1:** Summary of experimental and human data on relation between fat type and cancer.

In Vitro			In Vivo			Human Data			
Cell Line, Fat Specification	Outcome	Reference	Model, Fat Specification	Outcome	Reference	Cancer Type, Fat Specification	Outcome	Reference	
**SATURATED FAT**									
						All cancers	Positive association between high SFAs intake and cancer risk and mortality, respectively	[123,124]	
HER2/neu-positive breast cancer cells, PA	Induction of cell cycle delay and apoptosis	[151]	Spontaneous mammary tumours, C3H mice, diet supplemented with PA, SA, MA, and LaA, respectively	No effect of diet supplemented with PA, MA or LaA, respectively	[304]	Breast cancer, high SFAs intake	Positive association	[103,124]	
						Breast cancer, PA and SA intake	Positive association	[154]	
Hs578T human breast cancer cells, SA	Growth suppression via cell cycle inhibition	[161]				Breast cancer, PA intake	No association	[155]	
			NMU-induced mammary tumours, Sprague-Dawley rats, HFD rich in SA	Decreased tumour incidence and increased latency after SA supplementation	[161]				
SkBr3 breast cancer cells, LaA	Inhibition of proliferation, apoptosis stimulation	[167]							
MDA-MB-231 breast cancer cells, capric, caprylic and caproic acids	Cell growth inhibition and apoptosis stimulation	[178]	MDA-MB-435 xenografts, athymic mice, HFD rich in SA	Decreased incidence and multiplicity of tumours	[305]				
			Spontaneous mammary tumours, A/ST mice, HFD rich in SA	Growth suppression, increased tumour latency	[306]				
HCT-15 colon cancer cells, LaA	Apoptosis induction	[9]	Azoxymethane-induced colorectal cancer, F344 rats, HFD rich in SFAs	Increased incidence and multiplicity of colon tumours, induction of colonic inflammation	[307]	Colon cancer, SFAs intake	No association	[112]	
Caco-2 human colon cancer cells, LaA	Suppression of proliferation	[168]	HCT116 colorectal cancer xenografts, nude mice, HFD rich in PA	Tumour growth stimulation	[8]				
CT26 mouse colon cancer cells, LaA	Suppression of proliferation, increase in oxidative stress	[169]							
HCT-116 colorectal cancer cells, capric, caprylic and caproic acids	Cell growth inhibition, apoptosis stimulation	[178]							
Hep3B, SW480, SW620, AGS, BGC-823, HGC-27, 97H, and LM3 hepatocarcinoma cells, PA	Reduced cell proliferation, impaired cell invasiveness	[152]	LM3 hepatocarcinoma xenografts, athymic mice, PA (via gavage)	Tumour growth suppression	[152]				
PNT1A and PC3 prostate cancer cell lines, PA	Increased proliferation and migration	[147]	PC-3 prostate cancer xenografts, SCID mice, HFD rich in PA	Stimulated proliferation	[98]	Prostate cancer, SFAs intake	Positive association	[125]	
						Prostate cancer, PA intake	Positive association	[154]	
						Prostate cancer, PA intake	No association	[155]	
						Prostate cancer, MA intake	Positive association	[176]	
AsPC-1 pancreatic cancer cells, PA	Increased invasiveness	[148]	Nude mice, HPAF pancreatic cancer xenografts, HFD rich in SFAs	Increased tumour viability	[308]	Pancreatic cancer, SFAs intake, PA and SA intake	Negative association	[127]	
MIA PaCa-2, PANC-1 and CFPAC pancreatic cancer cells, PA, SA, LaA	Growth inhibition	[309]							
Gastric cancer cell lines, PA	Promotion of metastasis	[150]							
Oral carcinoma cell lines PA	Increased metastasis	[149]							
						Ovarian cancer, SFAs intake	Positive association	[158]	
							No association	[126]	
Ischikawa endometrial cancer cells, LaA	Inhibition of proliferation, apoptosis stimulation	[167]							
A-431 skin cancer cells, capric, caprylic and caproic acids	Cell growth inhibition, apoptosis stimulation	[178]							
**UNSATURATED FAT**									
**MUFAs**									
						Isocaloric replacement of SFAs with plant MUFAs	Decreased cancer mortality	[123]	
						Isocaloric replacement of animal MUFAs with plant MUFAs		[192]	
MCF-7 breast cancer cells, OA	Stimulation of proliferation	[180]				Breast cancer, olive oil consumption, highest vs lowest intake	Decreased risk	[191]	
	Suppressed growth and survival	[182]							
	Increased invasiveness	[184]							
MDA-MB-231, OA	Stimulation of growth and migration	[182]							
	Increased invasiveness	[184]							
BT-474 and SK-Br3 breast cancer cells, OA	Inhibition of Her-2/neu expression	[181]							
Caco-2 colon cancer cell line, OA	Growth promotion	[186]				Colon cancer, MUFAs intake	No association	[112]	
SGC 7901gastric carcinoma cells, OA	Suppressed growth and survival	[182]				GIT cancer, MUFAs intake	Decreased risk	[124]	
HGC-27 gastric carcinoma line, OA	Stimulation of growth and migration	[182]				GIT cancer, olive oil consumption, highest vs lowest intake		[191]	
MKN-45 and AGS gastric cancer cell lines, OA	Increased invasiveness	[185]							
						Prostate cancer, MUFAs intake	Positive association	[125]	
						Ovarian cancer, MUFAs intake	No association	[126]	
			HeLa cervical cancer xenografts, BALB/c mice, diet high in OA	Increased growth and metastasis	[188]				
						Basal cell carcinoma, MUFAs intake	Inverse association between intake and risk	[113]	
786-O renal cancer cells, OA	Increased invasiveness	[187]							
CAL27 and UM1 tongue squamous cell carcinomas, OA	Induction of apoptosis and autophagy	[189]							
**PUFAs**									
**ω-6 PUFAs**									
						Isocaloric replacement of SFAs with LA	Decrease in cancer mortality	[123]	
						Colon cancer, PUFAs intake	No association	[112]	
MDA-MB-231 breast cancer cells, LA	Promotion of migration and invasion	[212]	DMBA-induced mammary tumours, Sprague-Dawley rats, diet high in LA	Stimulation of DMBA-DNA adducts formation in mammary gland	[221]	Breast cancer, ω-6 PUFAs intake	No association	[110]	
						Breast cancer, higher dietary ω-3 PUFAs / ω-6 PUFAs ratio	Lower risk in Asian countries	[222]	
RKO and LOVO colon cancer cell lines, LA	Growth stimulation by low concentrations, grow inhibition by high concentrations	[209]	C57BL/6J mice, diet high in LA	Epigenetic alterations associated with colonic inflammation and cancer	[220]				
SW480 and SW620 colon cancer cells, LA	Decreased cell proliferation and viability	[210]							
AGS gastric adenocarcinoma cells, LA	Growth inhibition	[211]	CUM-2MD3 gastric carcinoma transplants, NCr-nu/nu mice, HFD rich in LA	Stimulation of invasion and metastasis	[218]				
			OCUM-2MD3 gastric carcinoma transplants, athymic nude mice, HFD rich in LA	Enhanced tumour growth and angiogenesis	[219]				
			Oral carcinomas induced by DMBA and betel quid extract, hamsters, high dietary ω-6 PUFAs / ω-3 PUFAs ratio	Tumour growth promotion	[216]				
MIA PaCa-2, PANC-1 and CFPAC pancreatic cancer cells, LA	Growth inhibition	[309]	HPAF pancreatic cancer xenografts, nude mice, HFD rich in ω-6 PUFAs	Increased tumour viability, stimulation of liver metastasis	[308]				
			Pancreatic neoplasia, KRAS transgenic mice, diet high in ω-6 PUFAs	Shortened tumour latency	[217]				
PC-3 and C4-2 prostatic cancer cells, AA and LA	Reduced cell proliferation and viability	[207]							
T98G glioblastoma cells, AA	Growth inhibition	[180]							
**ω-3 PUFAs**									
MCF-7 mammary cancer cells, ALA or ALA combined with EPA and DHA	Decreased viability	[223]	4T1 mammary tumour transplants, BALB/c mice, ω-3 PUFAs enriched diet	Decrease in proliferation and angiogenesis, stimulation of apoptosis	[243]	Breast cancer, highest ω-3 PUFAs intake vs lowest ω-3 PUFAs intake / high ω-6 PUFAs intake	Decreased risk	[254]	
MCF-7 cells, DHA	Reduced proliferation	[226]							
			LM3 mammary transplants, BALB/c mice, ALA enriched diet	Inhibition of tumour growth and metastasis	[244]	Breast cancer, fish ω-3 PUFAs intake	Decreased risk in Asian patients	[255]	
MDA-MB-231 cells DHA	Pyroptosis induction	[225]							
			DMBA-induced mammary tumours in offspring of rats fed with diet enriched with ALA or DHA and EPA, respectively, C57BL/6J mice	Tumour growth inhibition, reduced proliferation and stimulation of apoptosis	[245]				
HT-29 and CaCo-2 colorectal cancer cells, DHA	Decreased viability	[227]	Azoxymethane-induced colorectal cancer, F344 rats, HFD rich in ω-3 PUFAs	Decreased incidence and multiplicity of colon tumours in comparison with HFD rich in SFAs	[307]	Colorectal cancer, long-chained ω-3 PUFAs	Inverse association between intake and risk	[256]	
HCT-116 and Caco-2 cells, DHA	Anti-angiogenic activity	[228]							
HCT-116, HT-29, SW620, DLD-1 colorectal cancer cells, DHA	Decreased proliferation, enhancement of autophagy induced by oxaliplatin	[231]	HCT116 xenografts, BALB/c mice, DHA (i.p.)	Enhancement of autophagy induced by oxaliplatin	[231]				
			N-methyl phosphite nitrourea-induced colorectal cancer, rats, ω-3 PUFAs enriched diet	Tumour growth inhibition	[246]				
			Colorectal neoplasia, transgenic Apc ^Min/+^mice, dietary fish-oil ω-3 PUFAs	Decreased colorectal carcinoma growth	[247]				
			MC38 colorectal carcinoma, C57BL/6 mice, ω-3 PUFAs enriched diet	Tumour growth suppression	[248]				
MIA PaCa-2, PANC-1 and CFPAC pancreatic cancer cells, ALA, DHA, EPA	Growth inhibition	[308]	HPAF pancreatic cancer xenografts, nude mice, HFD rich in ω-3 PUFAs	Decreased tumour viability	[309]				
			Pancreatic carcinoma, KRAS mice, fish oil ω-3 PUFAs enriched diet	Tumour growth inhibition, reduced proliferation	[249]				
PANC-1 pancreatic cancer cells, DHA	Apoptosis induction	[232]							
SW1990, PANC-1 pancreatic cancer cells, EPA, DHA	Growth inhibition	[235]	PANC02 transplants, *fat-1* transgenic mice	Tumour growth inhibition, apoptosis induction	[235]				
MHCC 97-L metastatic hepatocarcinoma line	Decreased proliferation, DHA	[236]							
			Prostate carcinoma, *Pten*-knockout mice, diet enriched with ALA	Tumour growth inhibition	[250]	Prostate cancer risk, ω-3 PUFAs intake	No effect	[258]	
			Endometrial cancer xenografts, BALB/c mice, dietary ω-3 PUFAs	Tumour growth inhibition	[251,252]	Breast cancer, long-chain ω-3 PUFAs intake	Decreased risk in women with normal BMI	[257]	
SKOV-3 ovarian cancer line, EPA	Apoptosis induction	[237]				Ovarian cancer, PUFAs intake	No association	[126]	
SKOV3, A2780, HO8910 ovarian cancer cells, ALA, DHA	Decreased viability by ALA and DHA, inhibition of invasion and metastasis by DHA	[238]							
A549 non-small lung cancer cells, DHA	Inhibition of proliferation	[233,234]							
LLC murine lung cancer cells, DHA		[234]							
LA-N-1 neuroblastoma cells, DHA, EPA	Cell cycle arrest and induction of apoptosis	[239]	GL261 glioma transplants, *fat-1* transgenic mice	Induction of apoptosis and autophagy	[240]				
D54MG, U87MG and U251MG glioblastoma cells, DHA	Induction of apoptosis and autophagy	[240]							
G1a, ML-2, HL-60, THP-1, U937 and MOLM-13 acute myeloid leukaemia cell lines, DHA and EPA	Decrease in cell viability	[241]							
Molt-4 acute lymphoblastic leukaemia cells, DHA	Apoptosis induction	[242]							
**TFAs**									
**iTFAs**									
			Ehrlich tumour, CBA mice, dietary EA	Tumour growth promotion, decreased survival	[273]	Oestrogen-receptor negative breast cancer risk, serum level of iTFAs	Positive association	[302]	
CT-26 and HT-29 colorectal cancer cells, EA	Enhanced growth and metastasis	[270,271]				Colon cancer risk, TFAs intake	Positive association	[298,299]	
	Attenuation of 5-fluorouracil cytotoxicity	[271]	CT26 and HT29 transplants, BALB/c mice, dietary EA	Increased tumour growth and metastasis	[270,272]	Rectal cancer risk, fish TFAs intake	Positive association	[269]	
Caco-2 colorectal cancer cells, EA	No effect on growth	[186]							
CMT93 murine rectal carcinoma cell line, EA	Increased stemness, attenuation of 5-fluorouracil cytotoxicity	[271]							
						Stomach cancer risk, fish TFAs intake	Positive association	[269]	
						Prostate cancer risk, total TFAs intake	Positive association	[125]	
						Prostate cancer risk, fish TFAs intake	Negative association	[269]	
						Pancreatic cancer risk, vegetable TFAs intake	Negative association in men	[269]	
						Pancreatic risk, serum level of iTFAs	Positive association in men	[303]	
						Ovarian cancer risk, TFAs intake	Positive association	[301]	
SH-SY5Y neuroblastoma cells, EA	Growth inhibition, apoptosis induction	[274]				CNS cancer risk	Negative association in women	[269]	
LL2 murine lung cancer cell line, EA	Increased stemness, attenuation of 5-fluorouracil cytotoxicity	[271]				Lung cancer risk	Negative association in women	[269]	
						Non-Hodgkin lymphoma risk, vegetable TFAs intake	Negative association	[269]	
						Multiple myeloma, fish TFAs intake	Positive association		
						Bladder cancer risk, fish TFAs intake	Negative association	[269]	
**rTFAs**									
MCF-7 mammary carcinoma, VA	Inhibition of proliferation	[275]	Mammary tumour growth	Growth inhibition	Reviewed in [287]	Breast cancer risk, CLA intake	No association	[297]	
MCF-10A mammary cancer cells, VA	No effect	[276]	DMBA-induced mammary tumours in Sprague-Dawley rat offspring, maternal diet enriched with CLA	Decreased susceptibility to tumour induction	[291]	Post-menopausal breast cancer, rTFAs intake	Positive association	[269]	
MCF-7 and MDA-MB-231 cells, CLA	Growth inhibition	[278,281]							
	Potentiation of docetaxel effect	[279]							
MCF-7 cells, CLA-gemcitabine conjugate	Growth inhibition	[292]	MCF-7 xenografts, BALB/c mice, CLA-gemcitabine conjugate	Suppression of tumour growth	[292]				
SW480 colon carcinoma, VA	Inhibition of proliferation	[275]	CT29 xenografts, BALB/c mice, dietary CLA	Metastasis inhibition	[283]				
HCT-116 and HT-29 colorectal carcinoma, CLA	Isomer-dependent inhibition of proliferation, induction of apoptosis,	[282]							
			1,2-dimethylhydrazine-induced colon cancer, Sprague-Dawley rats, dietary CLA	Apoptosis induction	[310]				
SW480 colon cancer cells, CLA	Isomer-dependent effect on cell invasiveness	[283]							
			Azoxymethane-induced colon cancer, Sprague-Dawley rats, dietary CLA	Decrease in aberrant crypt foci formation, apoptosis induction	[311]				
			Azoxymethane and dextransodium sulfate-induced colorectal cancer, 57BL/6 mice, dietary CLA	Tumour growth promotion	[312]				
						Mouth/pharynx cancer risk, rTFAs	Positive association	[269]	
DU145 prostate carcinoma cells, CLA	Cell cycle inhibition	[285]	DU-145 transplants, SCID mice, dietary CLA	Inhibition of tumour growth and metastasis	[313]				
			R-3327-AT-1 transplants, Copenhagen rats, dietary CLA	No effect on tumour growth	[314]				
SKOV-3 and A2780 ovarian cancer cells, CLA	Isomer-dependent suppression of proliferation and migration	[284]							
RL 95-2 endometrial cancer cells, CLA	Apoptosis induction	[286]							
5-8F and CNE-2 human nasopharyngeal carcinoma	Inhibition of proliferation, induction of apoptosis	[277]							
B16-F10 melanoma, liposomes containing CLA and paclitaxel	Growth inhibition	[293]	B16-F10 melanoma transplants, C57BL6/N mice, liposomes containing CLA and paclitaxel (i.v.)	Tumour growth inhibition	[293]	Malignant melanoma risk, rTFAs intake	Negative association in women	[269]	
						Non-melanoma cancer risk, rTFAs intake	Positive association		
						Multiple myeloma risk, rTFAs intake	Negative association	[269]	
						Non-Hodgkin’s lymphoma risk, rTFAs intake	Positive association		

Abbreviations: AA—arachidonic acid; ALA—alpha-linolenic acid; CLA – conjugated linoleic acid; DHA—docosahexaenoic acid; DMBA—9,10-dimethyl-1,2-benz[a]anthracene; EA—elaidic acid; EPA—eicosapentaenoic acid; GIT—gastrointestinal tract; HFD—high-fat diet; LaA—lauric acid; LA—linoleic acid; MA—myristic acid; NMU—N-methyl-N-nitrosourea; MUFAs—monounsaturated fatty acids; OA—oleic acid; PA—palmitic acid; PUFAs—polyunsaturated fatty acids; SA—stearic acid; SFAs—saturated fatty acids; TFAs—trans fatty acids; iTFAs—industrially produced trans fatty acids; rTFAs—ruminant trans fatty acids; VA – vaccenic acid.

## 7. Targeting Lipid Metabolism in Cancer Treatment

Cancer cells display changes in nutrient uptake and metabolism to fulfil the demands of proliferating cells, including de novo lipogenesis. Thus, FAs uptake, biosynthesis, and lipolysis present promising targets for cancer intervention. In recent years, great attention has been given to a transmembrane protein CD36/SR-B2, also known as a fatty acid translocase, which mediates FAs uptake and utilisation [315]. CD36 was reported to be highly expressed in cancer cells and was associated with enhanced proliferation and migratory activity [316,317,318]. For example, CD36 overexpression was linked to increased invasiveness and metastasis of cervical cancer cells both in vitro and in vivo [188,318]. CD36 expression was also correlated with lower survival rates and overall poor prognosis of cancer patients [188,319,320,321]. Transforming growth factor beta (TGF-β) downregulated E-cadherin and upregulated CD36 and mesenchymal markers, which indicates the interaction between CD36 and TGF-β in the promotion of EMT in cervical cancer [318]. On the other hand, the lower expression of CD36 in comparison with normal tissue was found in samples of pancreatic adenocarcinoma, but low CD36 expression was associated with large tumour size and poor survival prognosis [322]. These contradictions warrant further research.

Another interesting target is SCD, which catalyses the transformation of SFAs into MUFAs, mainly OA [323]. Two isoforms, SCD1 and SCD5, have been identified in humans, with the one aforementioned being the most prevalent [324]. The upregulation of SCD1 has been observed in a wide range of cancer cells and was associated with cancer aggressiveness and poor outcomes for patients [325,326,327]. So far, many SCD1 inhibitors have been tested and showed anticancer properties in preclinical studies, but due to adverse effects observed in vivo, only a few have progressed to clinical trials, and then almost exclusively as candidates for the treatment of type 2 diabetes [328].

There are other targets in lipid signalling pathways [329], and potential intervention may include modulation of lipid droplets biogenesis and lipophagy [330], but the research on the mechanisms involved in these pathways and their role in the genesis and progression of cancer is still ongoing. Thus, the development of substances that would effectively disrupt lipid metabolism in cancer cells without toxic effects in normal cells remains a great challenge for experimental oncologists.

## 8. Conclusions

Detrimental effects of an HFD on human health arise predominantly from excess adiposity, particularly visceral adiposity and induction of inflammatory state. As the human data show, a mere cut on fat intake does not have an impact on cancer risk; it is the FAs spectrum of dietary fat that is significant. SFAs, particularly PA, have been long vilified for their detrimental health effects, but the current consensus of nutritionists is that saturated fat does not pose a risk when consumed in moderation within a well-balanced diet. The protective effects of ω-3 PUFAs against malignant transformation in human studies were not as eminent as indicated in preclinical reports; one of the reasons might be that the level of ω-3 PUFAs in human diets did not reach the doses effective in animal models and that their beneficial effects might be counteracted by ω-6 PUFAs. The role of iTFAs in carcinogenesis is unclear; preclinical studies are scarce, and despite the general opinion on their harmfulness, human studies are not consistent. Controversial results regarding the intake of rTFAs and risk of cancer were reported too, but it appears that some isomers of rTFAs may be beneficial. In general, human data on the link between different fat types and cancer risk show great heterogeneity, which might be attributed to various factors, including host genetics, intake of medication, and possible measurement error due to self-reported food consumption. Heterogenous results may also arise from differences in the content of various food contaminants/constituents which act as carcinogens, e.g., heavy metals, polycyclic aromatic hydrocarbons, nitrosamines, or naturally occurring diacetyl, or, on the other hand, from varying content of protective substances like polyphenols in fruits and vegetables. Further studies with credible methodologies are needed.

## Abbreviations

AA	Arachidonic acid
ALA	Alpha-linolenic acid
AMPK	Adenosine monophosphate-activated protein kinase
AR	Adrenergic receptor
ATF	Activating transcription factor
aP2	Adipocyte fatty acid-binding protein
CD36	Cluster of differentiation 36
CDK2	Cyclin-dependent kinase 2
CHOP	C/EBP homologous protein, DNA damage-inducible transcript 3
CI	Confidence interval
CLA	Conjugated linoleic acid
DHA	Docosahexaenoic acid
DMBA	9,10-dimethyl-1,2-benz[a]-anthracene
EA	Elaidic acid
EGFR	Epidermal growth factor receptor
EMT	Epithelial-mesenchymal transition
EPA	Eicosapentaenoic acid
EPIC	European Prospective Investigation into Cancer and Nutrition
ER	Endoplasmic reticulum
ERK1/2	Extracellular signal-regulated kinase 1/2
FAK	Focal adhesion kinase
FAs	Fatty acids
GIT	Gastrointestinal tract
GPR	G-protein-coupled receptor
GSK-3β	Glycogen synthase kinase-3 beta
HDAC	Histone deacetylase
HDACi	Histone deacetylase inhibitor
HFD	High-fat diet
HMGB1	High mobility group box 1 protein
HR	Hazard ratio
IARC	International Agency for Research on Cancer
IL	Interleukin
iTFAs	Industrially produced trans fatty acids
LaA	Lauric acid
LA	Linoleic acid
LPS	Lipopolysaccharides
MA	Myristic acid
MAPKs	Mitogen-activated protein kinases
MCP-1	Monocyte chemoattractant protein-1
MMP	Matrix metalloproteinase
mTOR	Mammalian target of rapamycin
MUFAs	Monounsaturated fatty acids
NF-κB	Nuclear factor kappa B
NMU	N-methyl-N-nitrosourea
NOX	Nicotinamide adenine dinucleotide phosphate oxidase
Nrf2	Nuclear factor erythroid 2-related factor 2
OA	Oleic acid
OR	Odds ratio
p21(CIP1/WAF1)	Cyclin-dependent kinase inhibitor 1
p27 (KIP)	Cyclin-dependent kinase inhibitor 1B
PA	Palmitic acid
PCNA	Proliferating cell nuclear antigen
PI3K	Phosphatidylinositol 3-kinase
PPAR	Peroxisome proliferator-activated receptor
PUFAs	Polyunsaturated fatty acids
Q	Quintile
RCT	Randomised controlled trial
RNS	Reactive nitrogen species
ROS	Reactive oxygen species
RVLM	Rostral ventral lateral medulla
RR	Relative risk
rTFAs	Ruminant trans fatty acids
SA	Stearic acid
SREBP	Sterol regulatory element-binding protein
SCD	Stearoyl-CoA desaturase
SFAs	Saturated fatty acids
STAT	Signal transducer and activator of transcription
T	Tertile
TFAs	Trans fatty acids
TGF-β	Transforming growth factor beta
TLR-4	Toll-like receptor 4
TMA	Trimethylamine
TMAO	Trimethylamine-N-oxide
TNFα	Tumour necrosis factor alpha
UPR	Unfolded protein response
VA	Vaccenic acid
XBP1	X-box binding protein 1
